# Nanosize effects in metal oxide-supported gold catalysts

**DOI:** 10.1039/d6sc04087j

**Published:** 2026-08-17

**Authors:** Hiroaki Tada, Shin-ichi Naya

**Affiliations:** a Institutes of Innovation for Future Society, Nagoya University Furo-cho, Chikusa-ku Nagoya Aichi 464-8603 Japan htada0409@gmail.com; b Environmental Research Laboratory, Kindai University 3-4-1, Kowakae, Higashi-Osaka Osaka 577-8502 Japan shinichi.naya@itp.kindai.ac.jp

## Abstract

CO oxidation by O_2_ in the air is a very important reaction from both scientific and practical aspects. The discovery that Au particles with a diameter of less than 5 nm supported on metal oxides (Au/MOs) exhibit extremely high catalytic activity for CO oxidation even at 203 K has led to the current remarkable development of Au nanocatalysts. Numerous experimental and theoretical studies have deepened our understanding of the reaction mechanism. The clue to clarifying the origin of this surprising catalytic reaction is to understand the nanosize effects of Au particles on the physical properties and reactivity of Au/MO catalysts. This perspective highlights various nanosize effects in Au/MO thermal catalysts. The basic part discusses, in order, the size control of Au nanoparticles (NPs) on MOs, the dimensional effect of Au particles, the quantum size effect of Au particles, the electronic Au NP-MO support interaction, and O_2_ activation on Au/MO catalysts. By integrating these considerations with the important findings reported so far on low-temperature CO oxidation over Au/MO catalysts, we present a Langmuir–Hinshelwood (LH) type mechanism involving reductive O_2_ activation *via* water-assisted electron transfer from the MO support to O_2_ through Au NPs (SAO-ET) as the key step. This LH mechanism rationally explains the remarkable experimental results of the Au/MO catalysts in low-temperature CO oxidation, including the effects of Au particle size, MO support, and water addition. Subsequently, the thermal stability of Au nanocatalysts is described, which is the most important issue for practical applications, focusing on the atomic-level interfacial bonding with the MO support. Finally, we summarize the conclusions and important future challenges.

## Abstract 2

Based on various nano-effects of Au NPs loaded on metal oxides, we proposed a LH mechanism involving water-mediated electron transfer from the MO to O_2_*via* Au NPs to comprehensively explain key findings regarding low-temperature CO oxidation.

## Graphical abstract

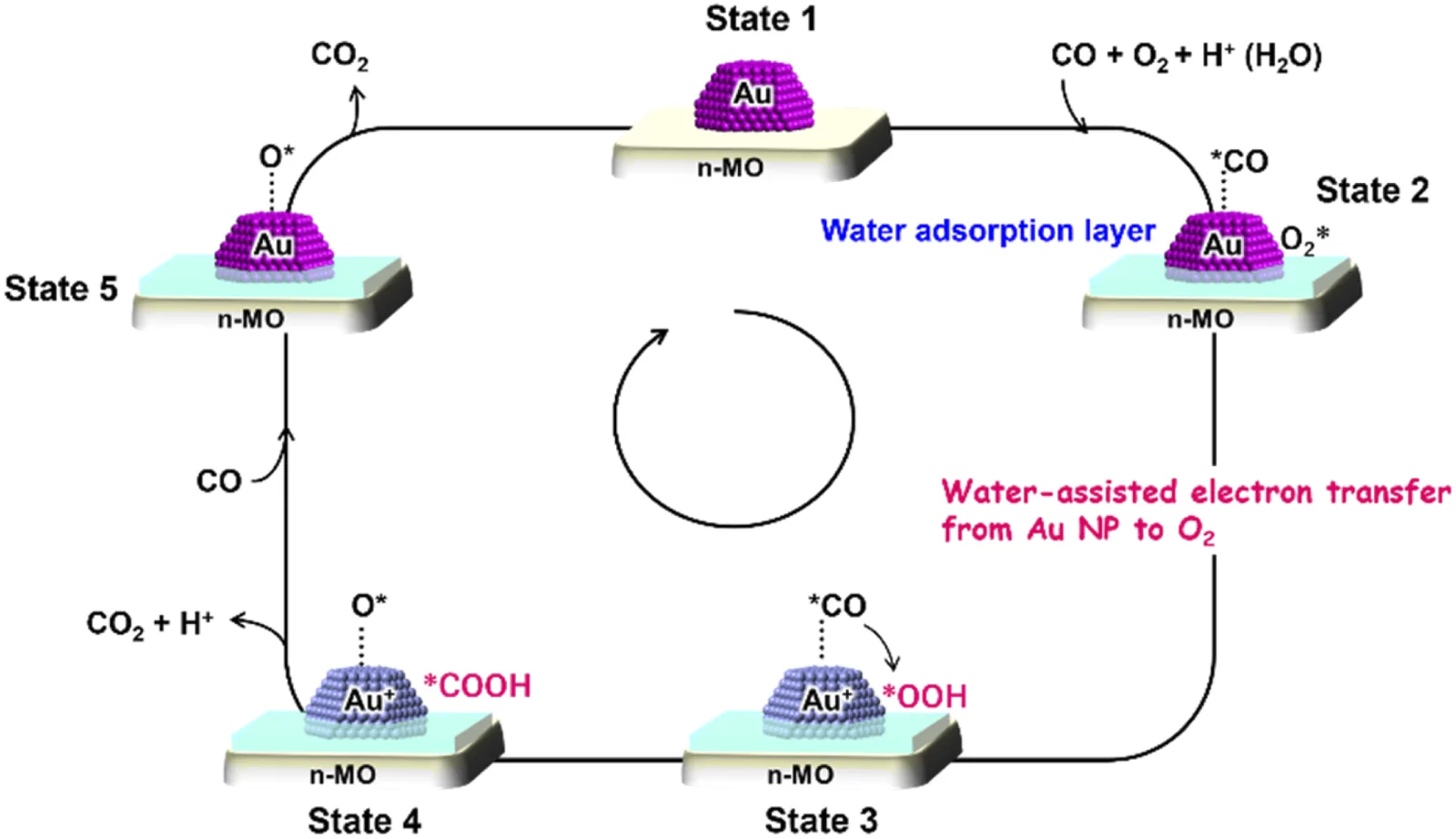

## 1 Introduction

In heterogeneous catalysis, CO oxidation is an important reaction for indoor air purification, automotive exhaust control, and fuel cells, while it is one of the best-known reactions and can be regarded as a benchmark system.^1^ In the late 1980s, Haruta and coworkers reported that Au particles with a diameter of less than 5 nm supported on metal oxides (Au/MOs) exhibited catalytic activity for CO oxidation at temperatures as low as 203 K, whereas MO-supported Pd and Pt catalysts typically require reaction temperatures above 373 K.^2^ This finding completely overturned the long-held belief that Au is chemically inert. Since then, intensive research has been conducted, demonstrating that Au nanocatalysts (NCs) exhibit high activity and selectivity for a variety of important chemical reactions.^3,4^ Among these, since the discovery, numerous experimental and theoretical studies have been conducted on CO oxidation over Au/MO catalysts, which have contributed significantly to a deeper understanding of the reaction mechanism.^3^ Comprehensive and excellent review papers on Au/MO catalysts have been published,^4,5^ and among these, a Langmuir–Hinshelwood (LH)-type mechanism with the periphery of Au NPs at the interface with the MO support (perimeter sites) as the active sites has been proposed for low-temperature CO oxidation on Au/MO catalysts. However, this alone cannot rationally explain all the key findings reported to date, and there has been no systematic discussion regarding thermal stability, which is the most important and difficult challenge for Au NCs in terms of practical applications.

In this paper, we demonstrate that incorporating an electrochemical perspective into the LH mechanism can rationally explain all of the important experimental results. Furthermore, the thermal stability of the Au/MO catalyst is discussed from the viewpoint of atomic-level interfacial bonding between Au NPs and the MO support. This perspective consists of a fundamental part on the Au nanosize effects in Au/MO systems (Section 2), a part on the catalytic activity of Au/MO systems for low-temperature CO oxidation (Section 3), and a part on the thermal stability of Au NPs on MOs (Section 4). Section 2 following the introduction describes the size control of Au nanoparticles (NPs) on MOs, dimensional effects of Au particles, quantum size effect of Au particles, electronic Au NP–support interaction, and O_2_ activation on Au/MO catalysts. Section 3 discusses Au particle size and MO support effects on the Au/MO-catalyzed low-temperature CO oxidation and the reaction mechanism. Section 4 deals with the thermal stability of Au/MO catalysts in terms of the interfacial bonding between Au NPs and the MO support. Finally, conclusions with some possible future challenges for the Au/MO catalysts are summarized in Section 5.

## 2 Fundamentals

### 2.1 Size control of Au NPs on MO supports

Because the activity of Au NCs depends on both particle size and the amount loaded, a technique for varying only the size in the range of 10 nm or less, while keeping the Au loading constant, is essential to investigate the size dependence of activity.

**Fig. 1:**
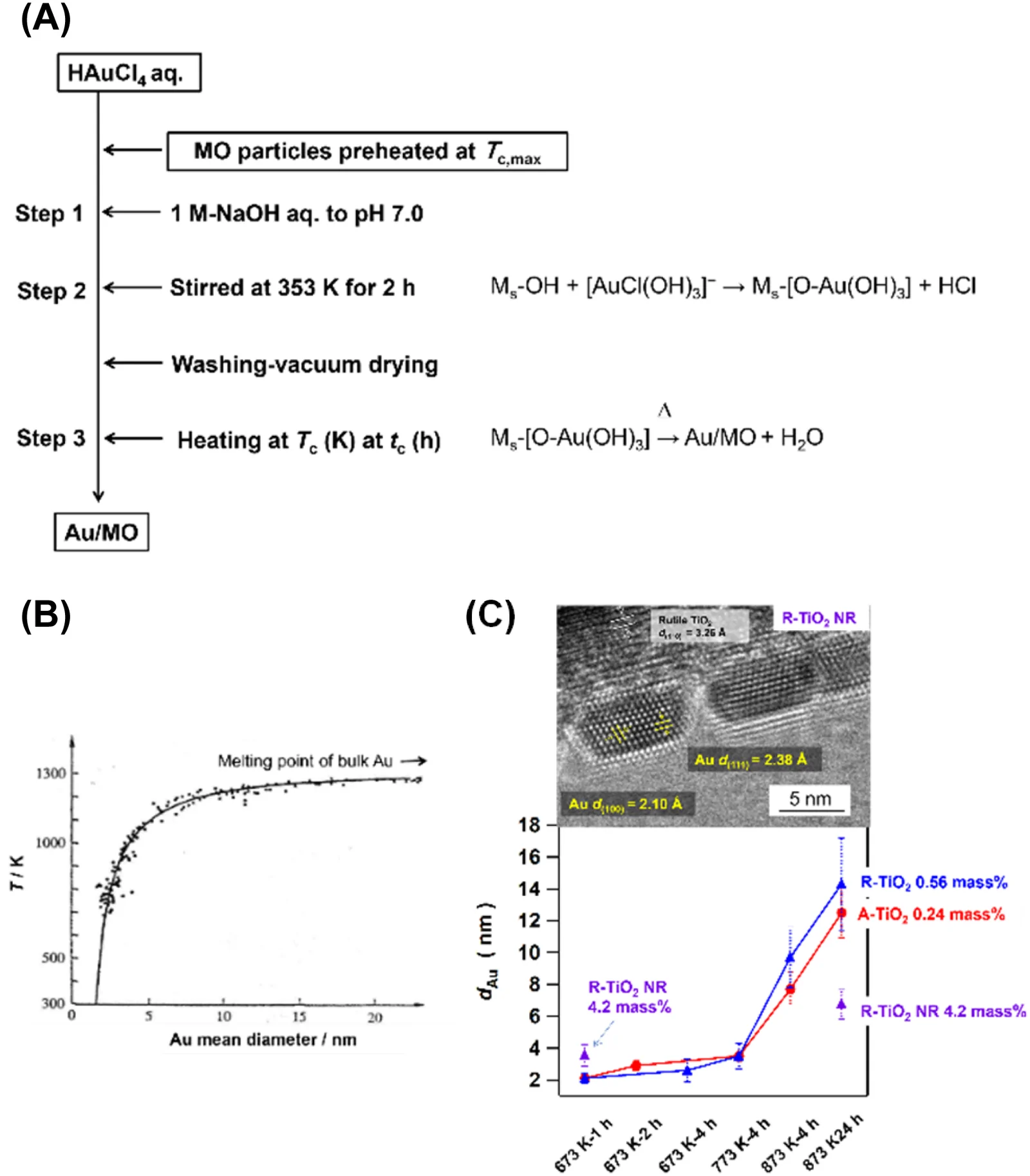
(A) Flowchart of the heating temperature-varied deposition precipitation (CTV-DP) process. (B) Particle size-dependence of the melting temperature of Au. (C) Relation between the mean Au particle size (*d*_Au_) and heating temperature (*T*_c_) for Au/anatase (A)-TiO_2_, Au/rutile (R)-TiO_2_, and Au/R-TiO_2_ nanorods (NRs) prepared by the CTV-DP method. The upper inset shows a high resolution-transmission electron micrograph (HR-TEM) of Au/R-TiO_2_ NRs. Panel (B) is reproduced from ref. 8, Copyright (1976), with permission from the American Physical Society.

Au NPs can be deposited on MO supports in a highly dispersed state by the deposition precipitation (DP) method developed.^6^ The DP process consists of neutralization of HAuCl_4_ solution (step 1), chemisorption of the resulting [Au(OH)_3_Cl]^−^ complex on MOs *via* ligand exchange (step 2), and Au NP formation by heating in the air (step 3) (Fig. 1A). It should be noted that in this DP process, the Au loading is determined in step 2.

In the nano-sized regime, the melting point of metal particles (*T*) decreases from their bulk value (*T*_0_) as their diameter (*d*_m_) decreases and the melting point depression (Δ*T* = *T*_0_ − *T*) increases inversely with *d*_m_ (eqn (1)).^7^

1 Δ*T* = 4*T*_0_*γ*/Δ*Uρd*_m_

where *γ* is the surface energy, Δ*U* is the heat of melting, and *ρ* is the density of the metal.

The melting point of Au particles decreases rapidly with decreasing *d*_Au_ in the region below 10 nm, dropping from 1337 K in the bulk to approximately 700 K at *d*_Au_ ≈ 2 nm (Fig. 1B).^8^ For metals, the relationship between the Tammann temperature (*T*_d_), where atomic diffusion in the material begins, and the melting point (*T*_m_) is *T*_d_ ≈ *T*_m_/3. Therefore, the *d*_Au_ can be controlled by changing heating temperature (*T*_c_) and time (*t*_c_) in step 3 of the DP method.^9^

The data for Au/anatase (A) and rutile (R) TiO_2_ prepared using this calcination temperature-varied (CTV)-DP method show that the *d*_Au_ can be controlled over a wide range from 2 nm to 14 nm with the Au loading maintained constant (Fig. 1C). Although this method can be applied to the formation of Au NPs on various MO supports, the catalytic activity of Au/MOs can also be affected by the change in the state of the MO support due to the difference in *T*_c_. To minimize this influence, it is desirable to heat-treat the MOs at a temperature above the maximum *T*_c_ before subjecting them to the CTV-DP method. Exceptionally, in the Au/R-TiO_2_ nanorod (NR) system (the upper inset in Fig. 1C), the increase in the *d*_Au_ with increasing *T*_c_ is much smaller than those of Au/A-TiO_2_ and Au/R-TiO_2_ systems, and the reason is discussed in Section 4.

Thus, by utilizing the thermal nano-effect of Au NPs, it is possible to form Au NPs with controlled mean sizes on various MO supports while maintaining a constant loading.

### 2.2 Dimensional effects of Au particles

At a constant Au loading, the total surface area of the highly dispersed hemispherical Au particles on the support (*S*_Au_) and the total perimeter distance of the Au particles at the interface with the support (*L*_P_) increase in proportion to 1/*d*_Au_ and 1/*d*_Au_^2^, respectively. For example, consider a system in which 2.5 mass% of Au particles are supported on an R-TiO_2_ surface in isolation. The *S*_Au_ of a system loaded with 2 nm *d*_Au_ particles increases by approximately one million times compared to the value when the same amount of one particle is loaded. On the other hand, the *L*_P_ per gram of Au/TiO_2_ reaches 4 × 10^6^ km at 5 mass% loading, which is equivalent to about 100 times the circumference of the Earth.

Therefore, in supported metal catalysts, when the periphery of the metal particles at the interface with the support (or perimeter sites) functions as active sites, reducing the size of the metal particles can be extremely effective in improving activity.

### 2.3 Quantum size and related effects of Au particles

In 1962, Kubo theoretically showed that discretization of energy levels can occur as metal particles become smaller (eqn (2)).^10^

2 *δ* = *E*_F_/*N*

where *δ* is the spacing between adjacent levels, *E*_F_ is the Fermi energy, and *N* is the number of atoms in the particle.

This quantum size effect has also been experimentally confirmed for Au particles on a support.

**Fig. 2:**
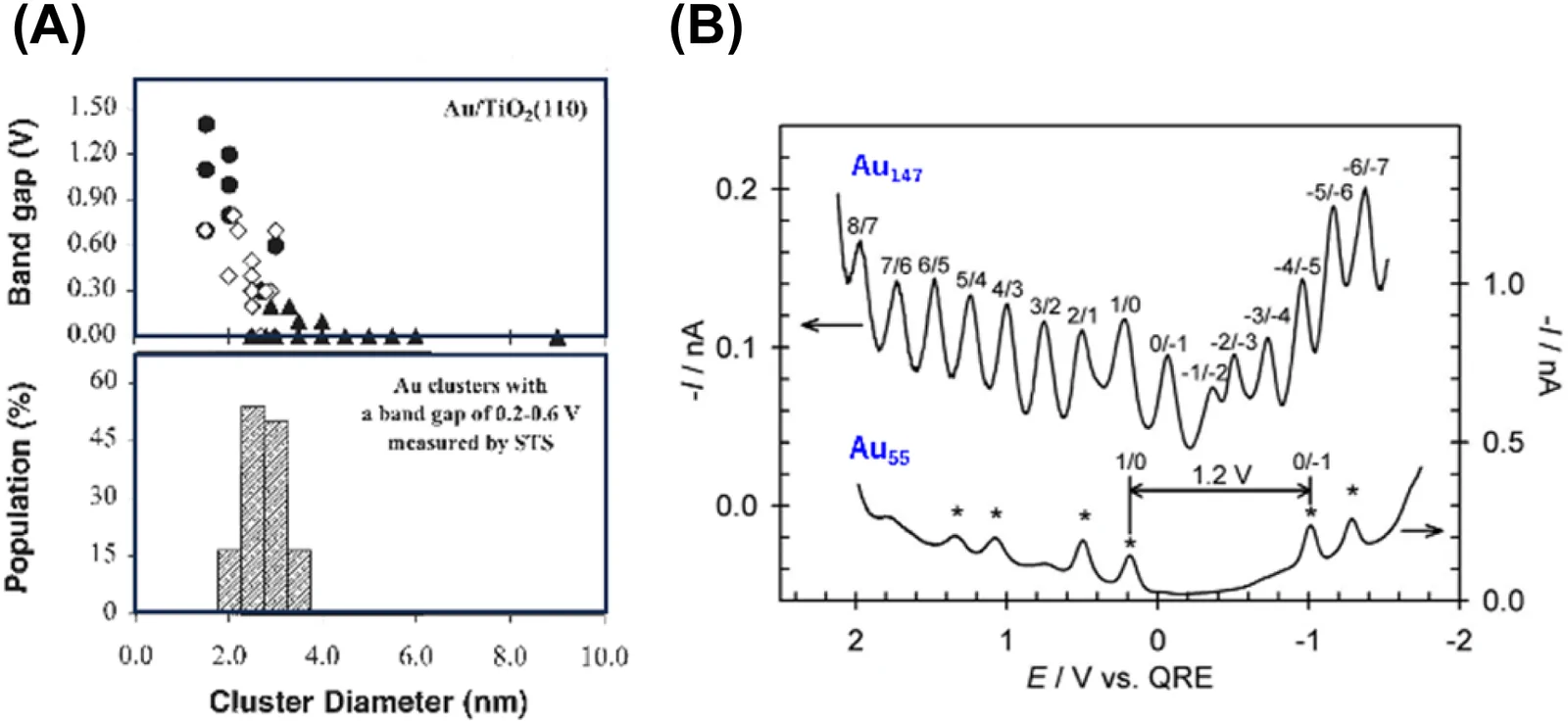
(A, upper) Cluster band gap measured by STS as a function of the Au cluster size supported on TiO_2_(110) − (1 × 1). The band gaps were obtained while the corresponding topographic scan was acquired on various Au coverages ranging from 0.2 to 4.0 ML. (●) Two-dimensional (2D) clusters; (h) 3D clusters with two atom layers in height; (▲) 3D clusters with three atom layers or greater in height. (A, lower) Relative population of the Au clusters (two atom layers in height) that exhibited a band gap of 0.2 to 0.6 V as measured by STS from Au/TiO_2_(110). (B) DPV responses for MPC solutions measured at a Pt microelectrode; as prepared 177 µM C6S-Au_147_ (upper) showing 15 high resolution QDL peaks and 170 µM C6S-Au_55_ (lower) showing a HOMO–LUMO gap. It can be seen that the as prepared solution contains a residual fraction of Au_55_ that smears out the charging response in *E* regions where QDL peaks overlap. The electrode potential scanned negative to positive. Panel (A) is reproduced from ref. 11, Copyright (1998), with permission from AAAS. Panel (B) is reproduced from ref. 12, Copyright (2003), with permission from the American Chemical Society.

Valden *et al.* formed Au clusters ranging in diameter from 1 to 6 nm on the (110) surfaces of single crystalline R-TiO_2_ in an ultrahigh vacuum.^11^ By means of scanning tunneling microscopy/spectroscopy (STM/STS), the authors revealed that a metal-to-nonmetal transition occurs as the cluster size is decreased below 3.5 nm in diameter and 1.0 nm in height (or 300 atoms per cluster) (Fig. 2A). Thus, the quantum size effect of supported Au NPs, *i.e.*, discretization of energy levels, appears for *d*_Au_ < ∼5 nm.

Thiol-protected Au clusters, so-called monolayer-protected clusters (MPCs), are known to behave as multivalent redox species. Quinn *et al.* studied MPC redox properties of hexanethiol capped Au clusters (C6S-Au_147_ and C6S-Au_55_) using electroanalytical techniques at a Pt microelectrode.^12^ The differential pulse voltammetry (DPV) response for C6S-Au_147_ shows 15 evenly spaced (Δ*V* ≈ 0.23 V) peaks characteristic of charge injection to the Au core (Fig. 2B, upper). On the other hand, in the DPV for the smaller C6S-Au_55_, a clear HOMO–LUMO gap of 1.2 V is observed with two and four reduction and oxidation peaks (Δ*V* ≈ 0.3 V), respectively, on either side of the band gap (Fig. 2B, lower). The discretization of the redox potential accompanying the successive addition and removal of electrons from metal clusters is primarily due to the electrostatic effect (Coulomb blockade) rather than quantum size effects.^13^

### 2.4 Electronic Au–MO support interaction

While most perfect crystals of MOs are inherently classified as insulators, they often exhibit semiconductor properties due to deviations from their stoichiometric composition and the presence of defects. In this case, by measuring the potential dependence of the differential capacitance with respect to the MO electrode and performing Mott–Schottky analysis, the flatband potential (*U*_fb_) and carrier density (donor density in the case of n-type and acceptor density in the case of p-type) can be determined. In this section, we discuss and analyse MOs that have been confirmed to possess n-type semiconducting properties by Mott–Schottky analysis.

When Au comes into contact with an n-type semiconducting metal oxide (n-MO), electrons transfer from the latter to the former with a large work function of 5.31 eV for Au until the Fermi energies (*E*_F_) of both are equal. In the bulk Au/n-MO junction system at the equilibrium, the *E*_F_ coincides with that of Au before contact due to the large density of states (DOS) of Au.

**Fig. 3:**
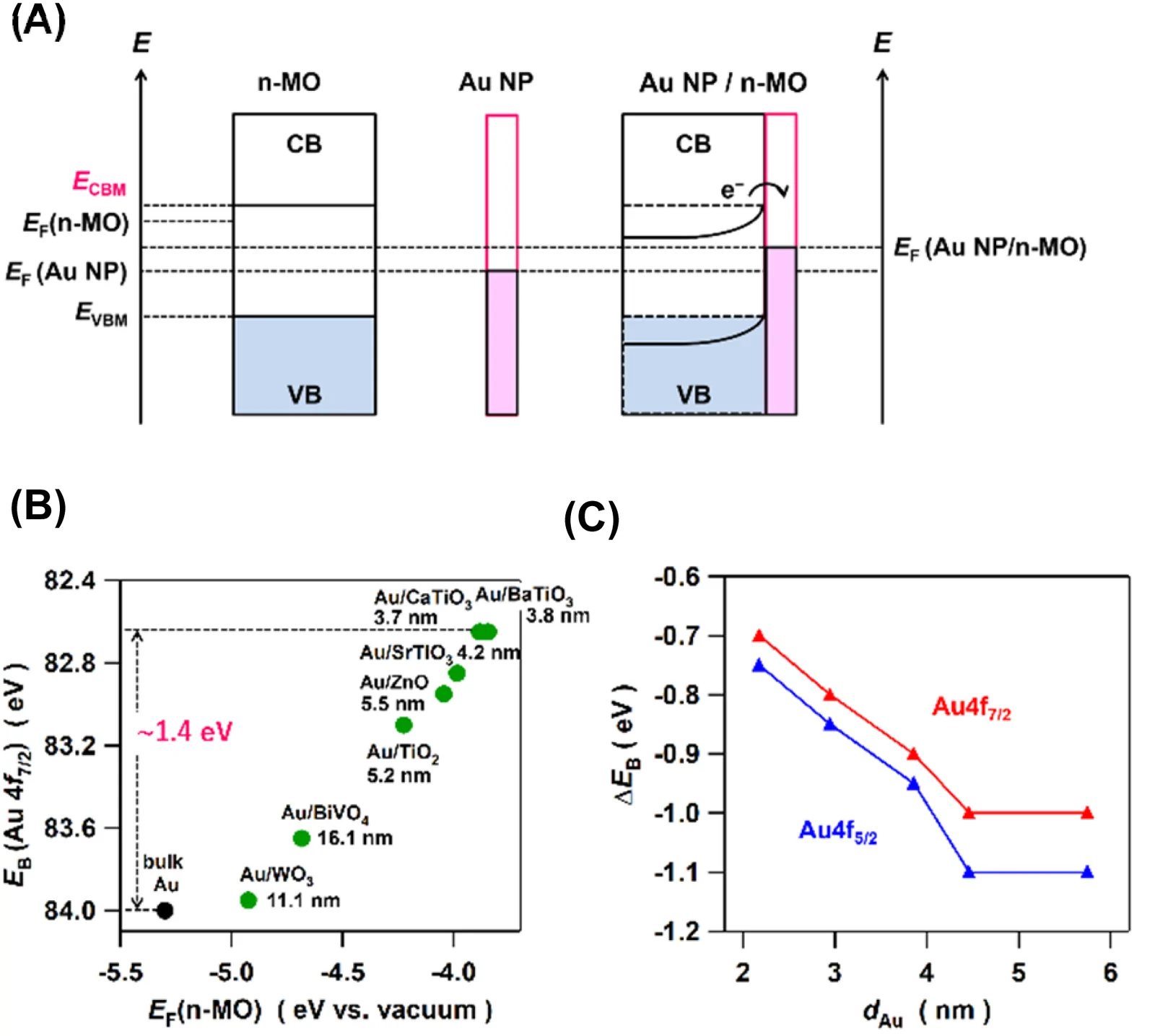
(A) Change in the Fermi energy (*E*_F_) of Au NPs before and after contact with n-MO. Anatase crystals were used as the TiO_2_ support. (B) Plots of the *E*_B_(Au 4f_7/2_) of Au/MOs *vs.* the *E*_F_ of the n-MO support. All *E*_F_ values in this figure were used without correction since the pH_pzc_ for some MOs was not available. (C) Δ*E*_B_ values for Au/TiO_2_ particles prepared by the CTV-DP method as a function of *d*_Au_. The samples were prepared by the CTV-DP method with the Au loading controlled to 4.2 mass%. Panel (B) is reproduced from ref. 15, Copyright (2016), with permission from the American Chemical Society.

In contrast, the *E*_F_ of the Au NP/n-MO junction system can increase compared to the value of Au before contact because the DOS of Au is significantly reduced (Fig. 3A). This means that in the Au NP/n-MO junction system, the electronic state of Au NPs can be controlled by the type of support. The *E*_F_(eV *vs.* vacuum) of various MOs can be estimated from the data on the *U*_fb_(V *vs.* SHE, pH 0)^14^ by using eqn (3), where SHE denotes the standard hydrogen electrode (SHE, pH 0).

3 *E*_F_(eV *vs.* vacuum) = −*e*(*U*_fb_ − 0.059 × pH_pzc_) − 4.44

where pH_pzc_ is the point of zero charge of the MO.

As evidence, there is a clear positive correlation between the *E*_F_ of the n-MO support and the Au4f binding energy (*E*_B_(Au4f_7/2_)) determined by X-ray photoelectron spectroscopy (XPS) for Au NP/n-MOs (Fig. 3B).^15^ According to the simple electrostatic potential model,^16^ the binding energy shift Δ*E*_B_(Au4f) can be affected by the initial effect (*k*Δ*q* + Δ*V*_M_) and the final effect (Δ*R*_ea_), where Δ*q* is the change in effective electronic charge on the Au atom with *k* being a constant, Δ*V*_M_ is the change in the Madelung potential, and Δ*R*_ea_ is the relaxation term (eqn (4)).

4 Δ*E*_B_(Au4f) = (*k*Δ*q* + Δ*V*_M_) − Δ*R*_ea_

The Δ*V*_M_ term represents the electrostatic interaction between a single Au atom and the charges on other atoms within the surrounding Au crystal lattice, while Δ*R*_ea_ arises from extra-atomic charge rearrangement (or relaxation) occurring in response to the core-level hole generated during the photoemission process. In the Au NPs loaded on different MO supports, the Δ*V*_M_ and Δ*R*_ea_ terms can be considered to remain largely unchanged, and thus, the difference of Δ*E*_B_(Au4f) can be mainly attributed to that of Δ*q* or the *E*_F_ of the hybrid system (Fig. 3A).

On the other hand, the effect of Au particle size on the *E*_B_(Au4f) of Au/TiO_2_ prepared by the CTV-DP method was examined by comparing the shift of *E*_B_(Au4f) of Au/TiO_2_ from the bulk Au value (Δ*E*_B_ = *E*_B_(Au/TiO_2_) − *E*_B_(bulk Au)). For all samples, the Δ*E*_B_ is negative, and its absolute value decreases as the size of the Au particles decreases in the range of *d*_Au_ < 4 nm (Fig. 3C). This result suggests that the number of electrons injected from TiO_2_ into the Au particles decreases with decreasing Au particle size, possibly due to the increasing repulsive force experienced by the excess electrons in the Au particles (Fig. 2B). In the Au/CaTiO_3_ and Au/BaTiO_3_ systems, the *E*_B_(Au4f_7/2_) decreases by about 1.4 eV compared to bulk Au (Fig. 3B), which is much larger than the variation with Au particle size (Fig. 3C).

Thus, the difference in *E*_F_ between the n-MO and the Au NPs before contact is the driving force for electron transfer from the former to the latter, whereas at *d*_Au_ < ∼4 nm, the electron capturing ability of Au NPs decreases due to the large Coulomb repulsion of excess charges in a small space (Fig. 2B). Notably, the latter effect is enhanced at smaller *d*_Au_ and in media with low dielectric constants, such as the gas phase.^13^

### 2.5 O_2_ activation on Au/MO catalysts

**Fig. 4:**
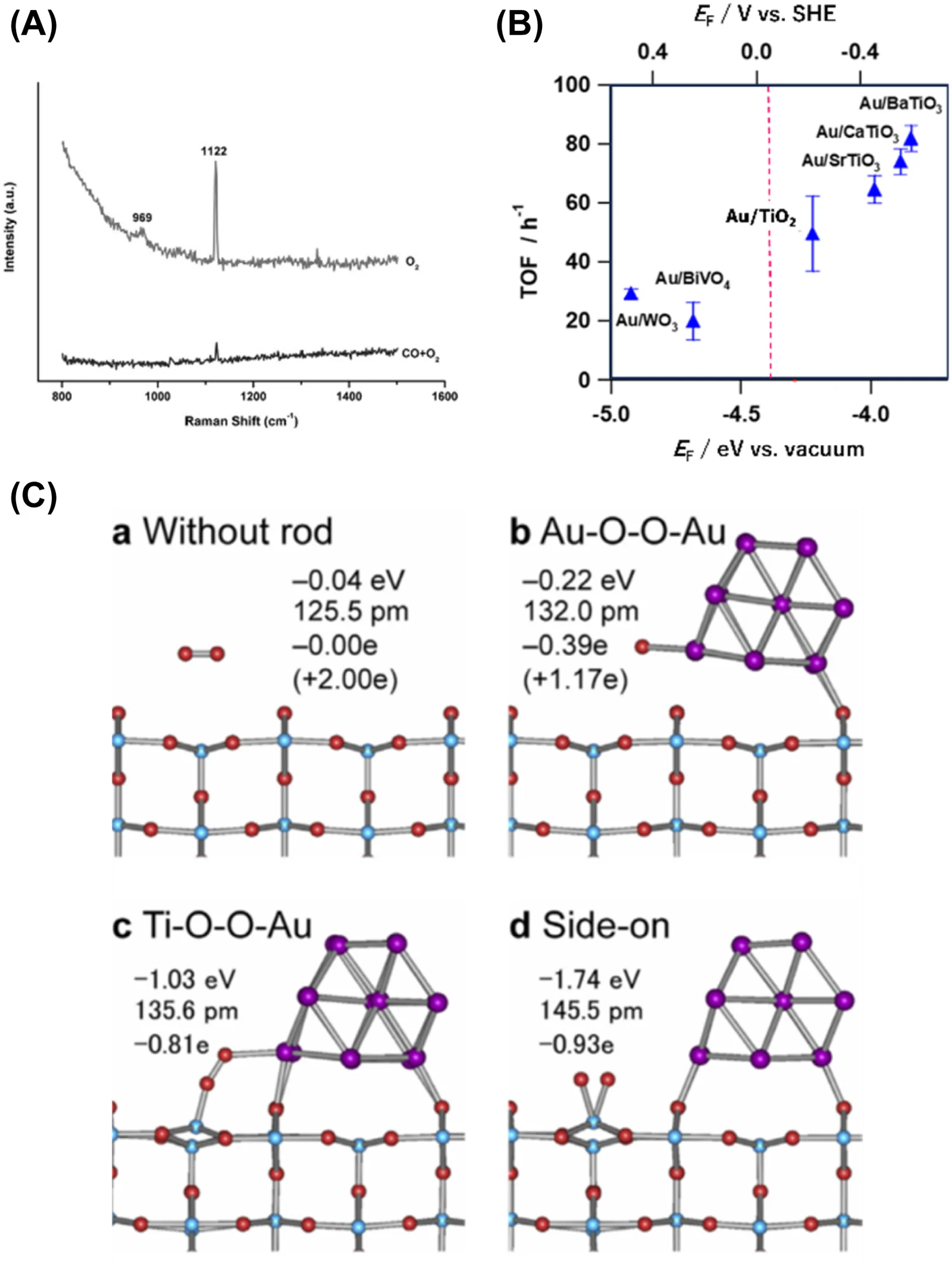
(A) Raman spectra of Au/FeTiO_2_ catalysts in O_2_ and CO + O_2_ atmospheres at 253 K. (B) Plots of TOF for H_2_O_2_ production from O_2_ saturated formic acid aqueous solution *vs. E*_F_ for the MO support. The dashed red line shows the standard electrode potential of *E*^0^(O_2_/HO_2_) (−0.046 V *vs.* SHE). As in Fig. 3B, all *E*_F_ values in this figure were also used without correction for pH_pzc_. The catalysts were prepared by the CTV-DP method. (C) Plots of TOF for H_2_O_2_ production from O_2_ saturated formic acid aqueous solution *vs. E*_F_ for the MO support. Panel (A) is reproduced from ref. 18, Copyright (2007), with permission from Wiley-VCH Verlag GmbH & Co. KGaA, Weinheim. Panel (B) is reproduced from ref. 20, Copyright (2019), with permission from the American Chemical Society. Panel (C) is reproduced from ref. 23, Copyright (2014), with permission from Elsevier.

CO oxidation is spin forbidden, because the total spins of the reactants (*S* = 1) and the product (*S* = 0) are different. As such, O_2_ activation is the key step in the CO oxidation over Au/MO catalysts.^17^ Corma and co-workers investigated the reactive oxygen species generated in the presence of O_2_ on the surfaces of Au/FeTiO_2_ and Au/TiO_2_ catalysts at 253 K using Raman spectroscopy.^18^ When Au/FeTiO_2_ is exposed to O_2_, two signals appear at 1122 cm^−1^ and 969 cm^−1^, which can be assigned to *η*^1^-superoxide species and peroxide adsorbed species, respectively (Fig. 4A, upper). Subsequent introduction of a mixture gas of CO and O_2_ almost eliminates these signals (Fig. 4A, lower). Similar results were obtained with the Au/TiO_2_ catalyst, although their signal intensities were weaker. Evidently, the superoxide and peroxide produced by the reduction of O_2_ on the Au/MO catalyst surface are the active species for CO oxidation.

In the reduction reactions on the Au/n-MO catalyst, the *E*_F_ of the n-MO support can be an important activity controlling factor in the catalytic reaction as well as in the photocatalytic reaction.^19–21^ O_2_ is reduced to H_2_O_2_ in aqueous formic acid at 298 K over Au/MO catalysts.^20^ The catalytic activity of Au/MOs is highly dependent on the *E*_F_ of the MO support, with the catalytic activity increasing with increasing *E*_F_ (Fig. 4B), and a similar relation was also obtained in the aerobic partial oxidation of alcohols.^15^ These results indicate that O_2_ is reductively activated on the Au/MO catalyst by electron transfer from the MO support to O_2_*via* Au NPs (SAO-ET), which was also evinced by Wei and co-workers by means of XPS spectroscopy, electron paramagnetic spectroscopy, and diffuse reflectance infrared spectroscopy.^22^ Furthermore, the use of MO supports with higher *E*_F_ would make the Au NPs more electron-rich, facilitating this reductive O_2_ activation and, consequently, providing higher catalytic activity.

Koga *et al.* performed density functional theory (DFT) calculations to gain insight into the adsorption sites, adsorption states, and activation mechanism of O_2_ on Au rod/rutile TiO_2_.^23^ When O_2_ is adsorbed on the five-coordinate Ti (Ti^5c^) site of the stoichiometric TiO_2_(110) surface, the adsorption energy of O_2_ is negligibly small (−0.04 eV) (Fig. 4C-a). Also, the O–O bond length is almost equal to its value in the gas phase (125.4 pm), and O_2_ remains in the triplet state without charge transfer from the surface. Then, O_2_ adsorption around the Au/TiO_2_ interface was examined. Only a small adsorption energy (−0.22 eV) was obtained for O_2_ adsorption on the two Au sites close to the perimeter sites (Fig. 4C-b). In contrast, O_2_ bridging and Au at the interface provide a large adsorption energy (−1.03 eV) (Fig. 4C-c). A much larger adsorption energy (−1.74 eV) is obtained for O_2_ adsorbed on the Ti^5c^ sites adjacent to the Au rod with a side-on mode (Fig. 4C-d). The longer O–O bond and larger negative charge indicate that this O_2_ is activated to the peroxide state. The spin polarization in the superoxide and peroxide states was negligible. Furthermore, it was shown that the O_2_ adsorption energy gradually decreased with increasing distance of the Ti^5c^ adsorption sites from the perimeter of Au NPs. These simulation results indicate that O_2_ is preferentially adsorbed on the interfacial Ti^5c^ and Au sites in a bridge mode and on the two Ti^5c^ sites around the Au rod in a side-on mode. In each case, the SAO-ET reductively activates O_2_ to generate superoxide and peroxide species, which is well consistent with the experimental results (Fig. 4A and B).

Thus, SAO-ET can lead to the reductive activation of O_2_ around the periphery of the Au NPs, and by lifting the spin-forbidden nature, CO oxidation can proceed smoothly.

## 3 Catalytic activity of Au/MOs for CO oxidation

### 3.1 Au particle size effects

**Fig. 5:**
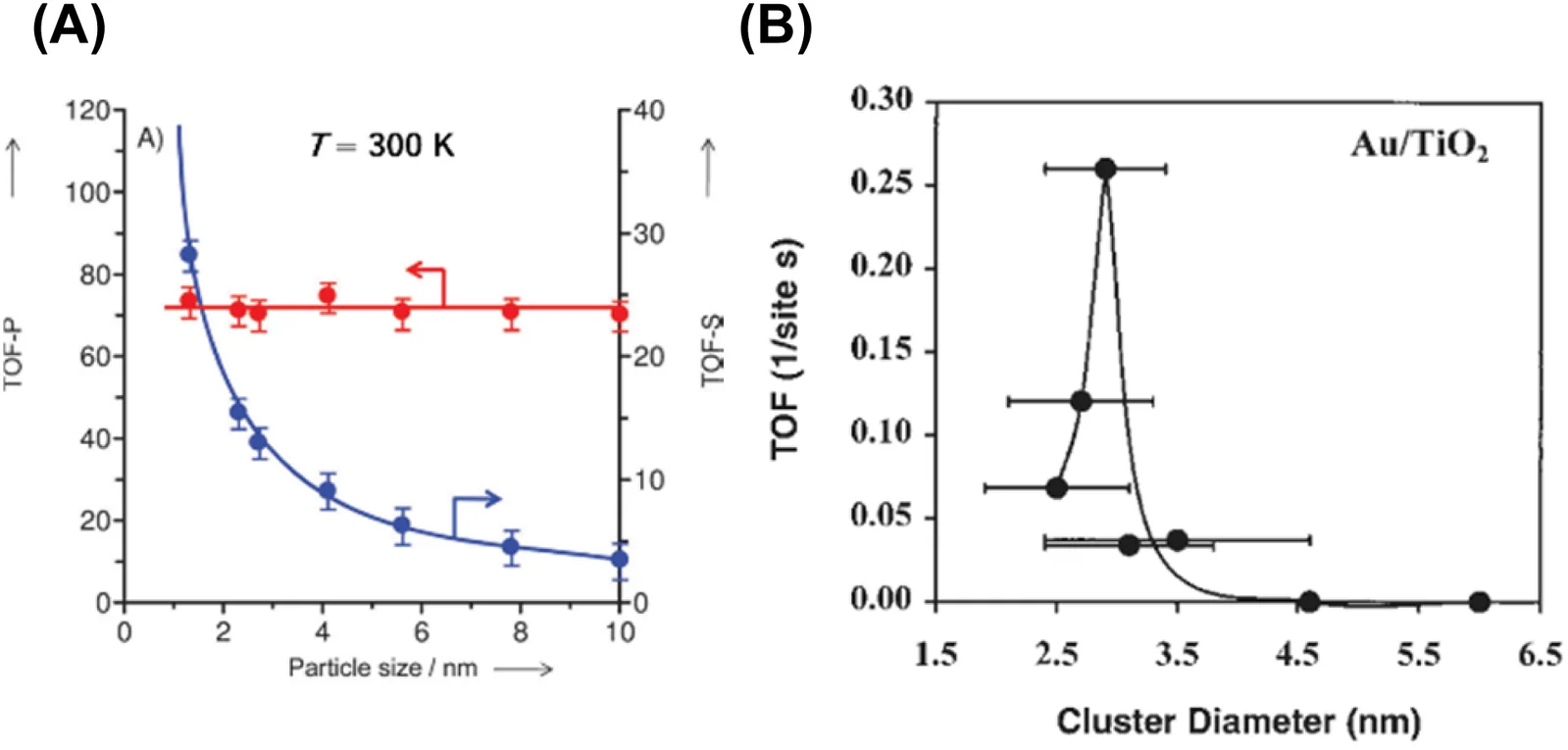
(A) TOFs for the formation of CO_2_ over one MLE of Au/TiO_2_-(110) as a function of the mean diameter of the Au particles at 300 K by normalizing the number of CO_2_ molecules formed per second to the total number of gold atoms at the perimeter interfaces (TOF-P) and by normalizing the number of CO_2_ molecules formed per second to the total number of exposed Au atoms at the gold particles (TOF-S). The oxidation of CO was performed in batch mode under 25 Torr of CO, 625 Torr of O_2_, and 0.1 Torr of H_2_O. (B) CO oxidation turnover frequencies (TOFs) at 300 K as a function of the average size of the Au clusters supported on a high surface area TiO_2_ support. The CO : O_2_ mixture was 1 : 5 at a total pressure of 40 Torr. The Au/TiO_2_ catalysts were prepared by the deposition-precipitation method, and the average cluster diameters were measured by TEM. Panel (A) is reproduced from ref. 24, Copyright (2011), with permission from Wiley-VCH. Panel (B) is reproduced from ref. 25, Copyright (1998), with permission from AAAS.

First, it is important to note that the *d*_Au_-dependence of the Au/MO catalyst for low-temperature CO oxidation differs significantly depending on whether or not H_2_O is present in the reaction system. In the presence of H_2_O, the turnover frequency per surface Au atom (TOF-S) for CO oxidation over Au/TiO_2_ catalysts at 300 K drastically increases at *d*_Au_ < 5 nm (Fig. 5A).^24^ On the other hand, in the absence of H_2_O, the catalytic activity of Au/TiO_2_ for CO oxidation at 300 K reaches its maximum value at approximately 3 nm for *d*_Au_ and decreases sharply below that value (Fig. 5B).^25^

Additionally, the dependence of catalytic activity on Au particle size provides information about the active sites. Fujitani and Nakamura studied the Au particle size-dependence of the catalytic activity of Au/TiO_2_ for CO oxidation and calculated the TOF-S and the total number of Au atoms at the perimeter sites (TOF-P).^24^ Unlike the *d*_Au_-dependency of the TOF-S, the TOF-P remains constant regardless of the *d*_Au_ (Fig. 5A). Also, the Arrhenius plots of CO oxidation over the Au/TiO_2_ catalyst provided an activation energy of 34.0 kJ mol^−1^ in the low temperature range (<320 K) and a value of 2.0 kJ mol^−1^ in the high temperature range (>320 K). Obviously, the reaction mechanism of Au/TiO_2_-catalyzed CO oxidation is different at low and high temperatures, and the reaction at low temperatures is strongly suggested to occur at the perimeter sites of Au NPs. These conclusions were further supported by the DFT calculations (Section 2.5).^23^

### 3.2 MO support effects

**Fig. 6:**
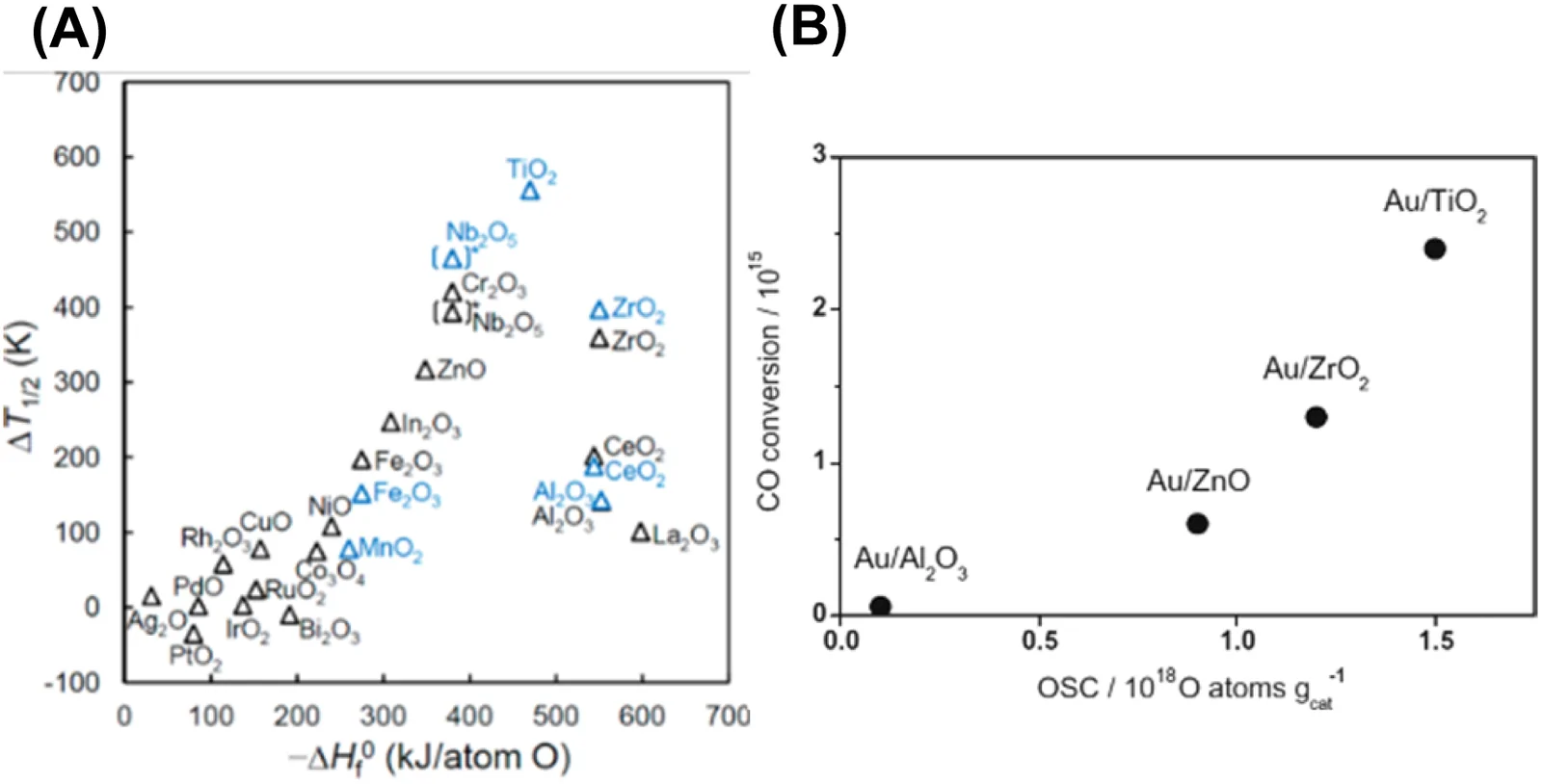
(A) Plots of the catalytic activity of Au/n-MOs (Δ*T*_1/2_) for Au/MO-catalyzed CO oxidation and −Δ*H*_f_^0^ of the MO support. Reaction conditions: catalyst of 150 mg, reactant gas is 1 vol% CO in air, flow rate of 50 mL min^−1^, SV 20000 h^−1^ mL g^−1^, moisture content of 50−200 ppm. (B) Amounts of CO molecules converted into CO_2_ in a steady state during simultaneous pulses of CO in Ar and O_2_ in Ar at 393 K as a function of the oxygen storage capacity (OSC) of Au/MOs. Panel (A) is reproduced from ref. 26, Copyright (2016), with permission from Elsevier B.V. Panel (B) is reproduced from ref. 29, Copyright (2010), with permission from Elsevier B.V.

The MO support also has a significant effect on the catalytic activity of Au NPs. It is generally recognized that Au/reducible MOs (TiO_2_, Fe_2_O_3_, and Co_3_O_4_) exhibit higher activity than Au/non-reducible MOs (SiO_2_, Al_2_O_3_, and MgAl_2_O_4_).^5^ Fujita *et al.* thoroughly investigated the support effect on the catalytic activity of Au/MOs for CO oxidation using various MO supports in the presence of H_2_O.^26^ They measured the temperature at 50% conversion of CO to CO_2_ (*T*_1/2_) in unmodified MO and Au/MO catalyst systems. The plots of *T*_1/2_*vs.* the standard enthalpy of formation per oxygen atom of MO (Δ*H*_f_^0^) in the MO and Au/MO systems were both scattered over a wide range. Meanwhile, the Δ*T*_1/2_ (=*T*_1/2_(MO) − *T*_1/2_(Au/MO)) *vs.* Δ*H*_f_^0^ plot shows a volcano-shaped curve (Fig. 6A). Therefore, the use of MOs with an appropriate metal–oxygen bond energy (300–500 kJ per atom O) as the support is effective for O_2_ adsorption and activation on the Au/MO catalyst, resulting in a significant improvement in catalytic activity for CO oxidation.

Based on the results of temporal analysis of products (TAP) and CO–O_2_ multipulse experiments, Widmann and Behm proposed an Au-assisted Mars-van Krevelen (MvK) mechanism for CO oxidation over Au/MO catalysts at temperatures above 353 K.^27^ which was further supported by DFT calculations.^28^ In this mechanism, CO adsorbed on Au NPs reacts with the surface lattice oxygen around the perimeter sites to generate CO_2_ and surface oxygen vacancies (V_o_s) in the MO lattice, and the surface lattice oxygen is reproduced by dissociative adsorption of O_2_ onto the V_o_s. In the CO oxidation, the catalytic activity of Au/MOs increases with increasing oxygen storage capacity (OSC) of the MO support (Fig. 6B).^29^

### 3.3 Reaction mechanism of low-temperature CO oxidation

Although it is widely recognized that the CO oxidation reaction over Au/MO catalysts at high temperatures follows the Au-assisted MvK mechanism, it does not hold for the low-temperature reaction that is of more interest to us because the lattice O desorption is unlikely at temperatures below 320 K. So far, for the low-temperature CO oxidation on Au/MO catalysts in the presence of H_2_O, an LH mechanism has been proposed that uses the perimeter sites as catalytically active sites.^30,31^

**Fig. 7:**
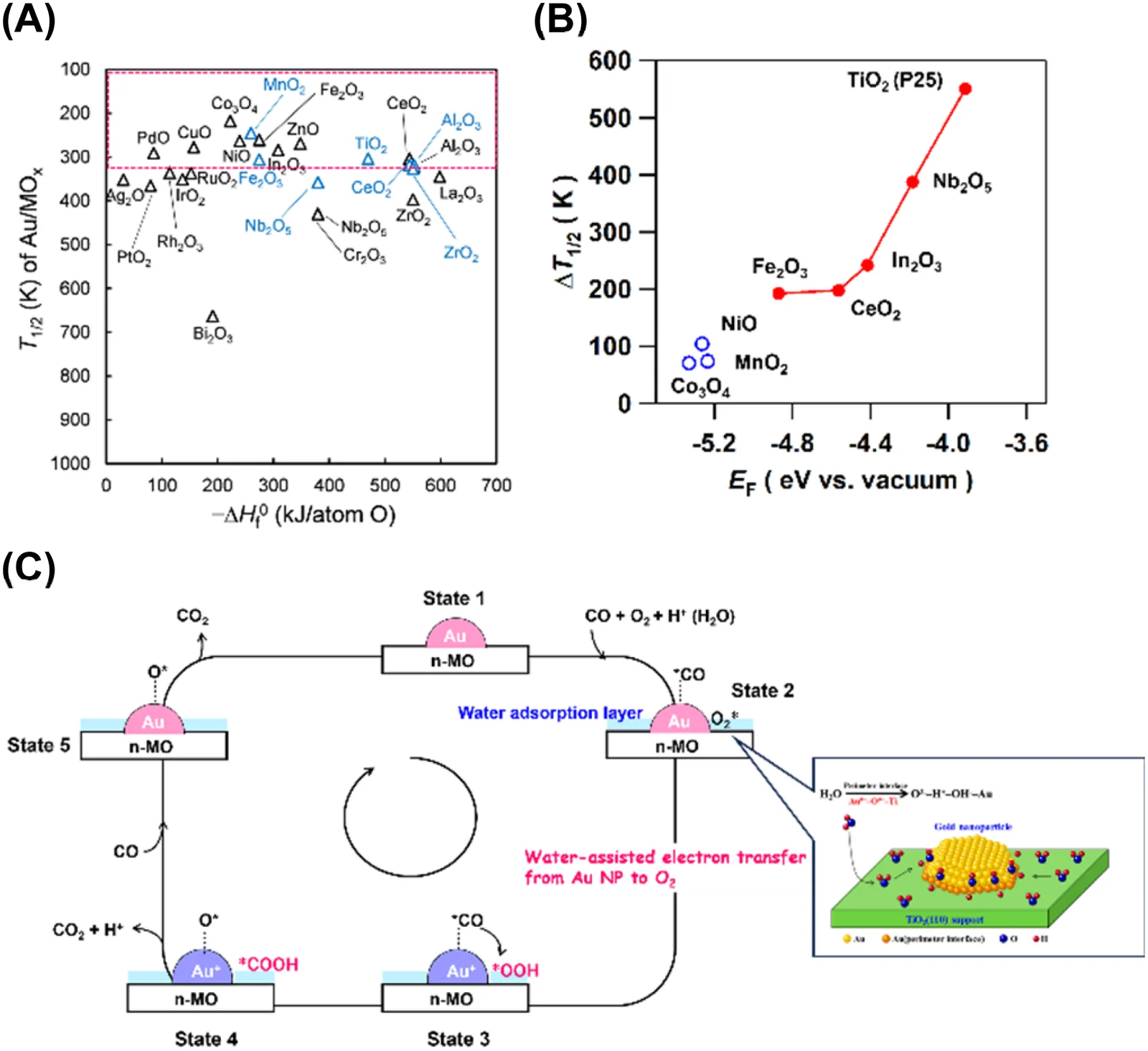
(A) Plots of the temperature of 50% conversion of CO to CO_2_ in the Au/MO systems (*T*_1/2_) for CO oxidation and the −Δ*H*_f_^0^ of the MO support. Reaction conditions: catalyst of 150 mg, reactant gas is 1 vol% CO in air, flow rate of 50 mL min^−1^, SV of 20000 h^−1^ mL g^−1^, moisture content of 50–200 ppm. (B) Relation between the catalytic activity of Au/MOs (Δ*T*_1/2_) and the *E*_F_ of the MO support. Data for n-type and p-type MOs are shown by filled red circles and hollow blue circles, respectively. The Δ*T*_1/2_ and *E*_F_ (or flatband potentials) values used were those reported in ref. 24 and 13, respectively. All *E*_F_s were calculated from eqn (2) using the pH_pzc_ of each MO. (C) The LH mechanism with water-assisted SAO-ET as the key step for low-temperature CO oxidation over Au/MO catalysts. The Au NPs shown in pink and purple represent electron-rich and electron-poor states, respectively. Panel (A) is reproduced from ref. 26, Copyright (2016), with permission from Elsevier B.V. The inset in panel (C) is reproduced from ref. 37, Copyright (2020), with permission from the American Chemical Society.

To gain insight into the mechanism of low-temperature CO oxidation over Au/MO catalysts, we analysed the relationship between the catalytic activity of Au/MOs and the *E*_F_ of MO supports. In this case, the Δ*T*_1/2_ data for MO-supported Au NPs were used among the Au/MO samples with *T*_1/2_ below 320 K (the region enclosed by the red dotted square in Fig. 7A). A clear positive correlation is observed between Δ*T*_1/2_ and the *E*_F_, regardless of whether the MO carrier is n-type or p-type (red filled circles and blue hollow circles, respectively, in Fig. 7B). Consequently, the catalytic activity for low-temperature CO oxidation is governed by the SAO-ET, as in the aerobic oxidation of alcohols over the Au/n-MO catalyst.^32^

Here, we present an LH mechanism of low-temperature CO oxidation over Au/MO catalysts incorporating water-assisted SAO-ET as a key step (Fig. 7C), which should explain the following important results: the catalytic activity drastically increases at *d*_Au_ < 5 nm (result 1); Au/MOs exhibit much higher catalytic activity than Pt/MOs (result 2);^33^ Au/n-MOs exhibit higher activity than Au/insulating MOs and Au/p-MOs (result 3). The presence of H_2_O significantly increases the reaction rate,^24,30,34,35^ whereas an excess of H_2_O conversely decreases it^36^ (result 4); the catalytic activity steeply decreases at *d*_Au_ < 2.5 nm in the absence of H_2_O (result 5).

In step 1, electron transfer from the n-MO carrier to Au makes the Au particles electron-rich. In step 2, when CO, O_2_, and H_2_O are introduced into the reaction system, CO and O_2_ are preferentially adsorbed on the surface of the Au NPs (*CO) and near the periphery of the Au NPs (O_2_*), respectively.^23^ Also, Fujitani *et al.* studied the reactivity of H_2_O on the Au/TiO_2_(110) surface using a temperature-programmed desorption technique, indicating that water adsorbed on the surface can dissociate at the perimeter sites (the inset in Fig. 7C).^37^ In step 3, subsequent electron transfer occurs from electron-rich Au NPs to O_2_*, generating *O_2_^−^ and *OOH,^31,35,38^ the latter being a coupling product of *O_2_^−^ and H^+^ provided by H_2_O.^31^ In step 4, *OOH and *CO react at the perimeter sites to yield O* and *COOH. In step 5, *COOH is decomposed into CO_2_ and H^+^, while electron-rich Au NPs are simultaneously regenerated. Finally, another *CO reacts with O* to produce CO_2_, and the catalytic cycle is completed.

According to this scheme, result 1 can be attributed to the sharp increase in *L*_p_, *i.e.*, the number of perimeter reactive sites, for *d*_Au_ < 5 nm.^39^ Result 2 is explained in terms of the adsorption energy of CO on Pt (1.26 ± 0.04 eV) much larger than that on Au (0.4 eV),^40^ which results in an increased activation energy,^23^ and/or more difficult diffusion of CO from the Pt NP surface to the perimeter sites. Result 3 is obtained because electron transfer from unreducible insulators as well as p-MOs with low *E*_F_ to the Au NPs, which is necessary for reductive O_2_ activation, is difficult. Result 4 can stem from the enhanced electron transfer from electron-rich Au NPs to O_2_* by coupling with H^+^ transfer, since the electrode potential of *E*^0^(O_2_/HO_2_) (−0.046 V *vs.* SHE) compared with *E*^0^(O_2_/O_2_^−^) (−0.33 V *vs.* SHE). Furthermore, the suppression of electron transfer from the MO to Au NPs due to a decrease in the electron-trapping ability of extremely small Au particles (Fig. 2B and 3C), which becomes more pronounced as the dielectric constant of the medium surrounding the Au/MO catalyst decreases, may be the cause of result 5. Furthermore, it can be inferred that the presence of excess water in the reaction system reduces the amount of O_2_ adsorbed on the catalyst surface due to the low solubility of O_2_ in water, thereby rather lowering the activity. Thus, the results 1–5 can be rationalized by the LH reaction mechanism with water-assisted SAO-ET as a key step. Exceptionally, Koroidov and co-workers recently demonstrated that low-temperature CO oxidation can proceed over Au/γ-Fe_2_O_3_*via* the MvK mechanism in the presence of H_2_O.^41^

## 4 Thermal stability

Au NPs are prone to aggregation and sintering at *d*_Au_ < ∼5 nm (Fig. 1B), while the activity of Au/MO catalysts decreases rapidly with increasing Au particle size in the range of *d*_Au_ > ∼5 nm (Section 3.1). Therefore, improving the thermal stability of Au NPs during the reaction is a crucial challenge for Au/MO catalysts. Metal–support interactions can play an important role not only in the electronic tuning of Au NPs (Section 2.4.) but also in their thermal stabilization on the support.

**Fig. 8:**
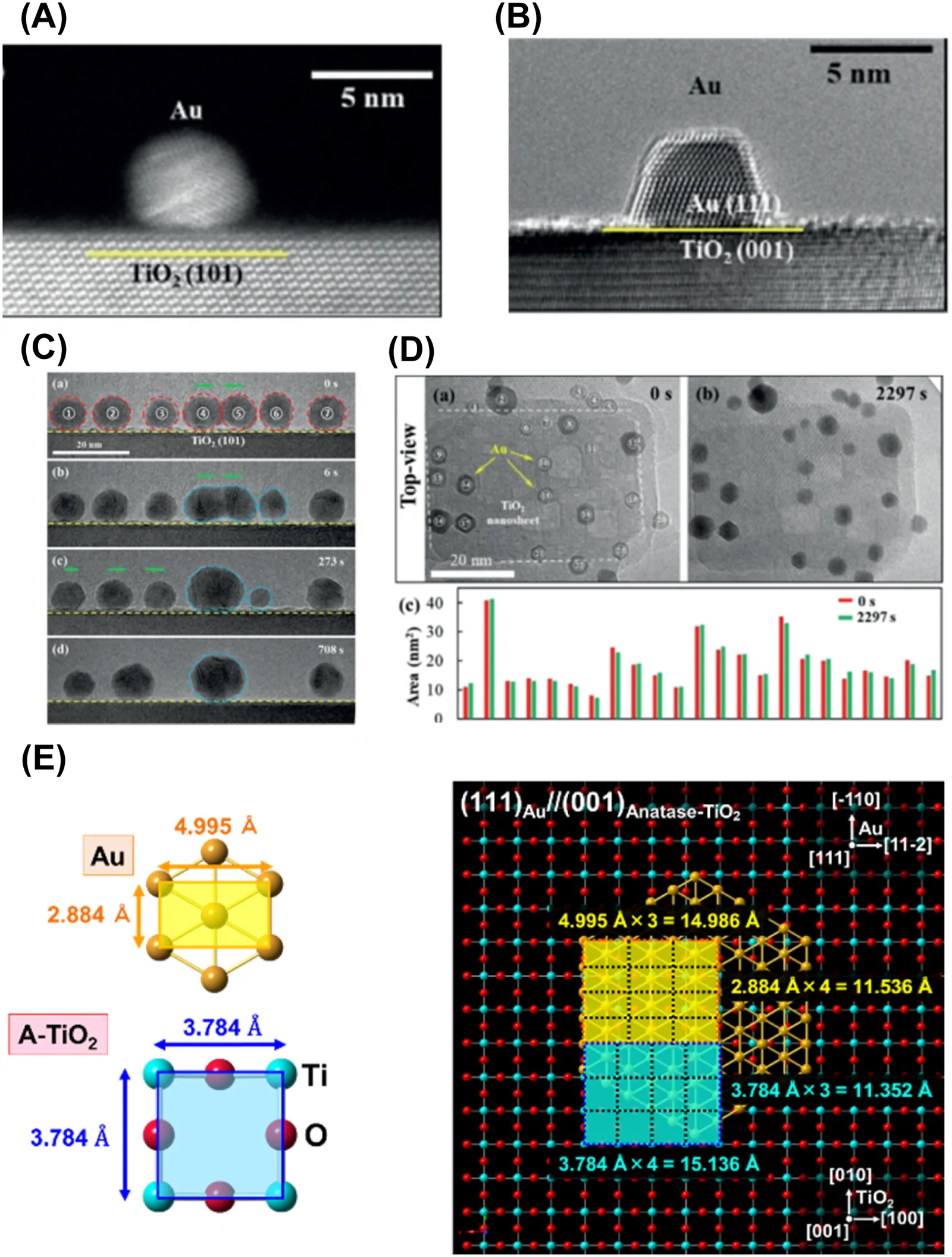
(A) High-magnification HAADF-STEM image of the typical interface between Au NPs and the TiO_2_ (101) surface. (B) HR-TEM image of the typical interface between Au NPs and the A-TiO_2_ (001) surface. (C) Sequential TEM images showing the sintering behavior of Au NPs on the A-TiO_2_(101) surface. The images were acquired at about 773 K and the oxygen pressure is about 5 × 10^−2^ Pa. (D) Sequential TEM images showing the sintering behavior of Au NPs on the A-TiO_2_(001) surface acquired under the same conditions as in (C). (E) An interface junction model with the (111)_Au_//(001)_A-TiO_2__ orientation. Panels (A–D) are reproduced from ref. 42, Copyright (2018), with permission from Wiley-VCH Verlag GmbH & Co. KGaA, Weinheim. Panel (E) is reproduced from ref. 43, Copyright (2024), with permission from the Royal Society of Chemistry.

Zhu, Gao, Wang, and co-workers investigated the effect of A-TiO_2_ facets on the sintering of Au NPs by means of *in situ* transmission electron microscopy (TEM).^42^ A typical high angle annular dark-field scanning TEM (HAADF-STEM) image of the Au-TiO_2_(101) interface shows that a nearly spherical Au NP with a diameter of approximately 4.8 nm is formed on the TiO_2_(101) surface (Fig. 8A). The contact area between the Au NP and TiO_2_ is small, and the interfacial length-to-diameter ratio (*I*_L/D_) ranged from 0.44 to 0.58. Meanwhile, in the high-resolution (HR)-TEM image of a typical Au/TiO_2_(001) sample, a faceted Au NP having a larger contact area with the TiO_2_ support is observed (Fig. 8B). The *I*_L/D_ ratios ranged from 0.80 to 0.96, which were significantly larger than those of the Au/TiO_2_(101) sample.

*In situ* TEM images of the Au/TiO_2_(101) sample during heating at 773 K in the presence of O_2_ clearly show that the sintering of Au NPs progresses with increasing heating time due to the Ostwald ripening (OR) and/or particle migration and coalescence (PMC). After 708 s of heating, the Au particle size increases, while the number decreases (Fig. 8C). In contrast, sintering is hardly observed in the Au/TiO_2_(001) sample even after 2297 s of heating (Fig. 8D). Apparently, the sintering of Au NPs is strongly influenced by the crystal plane on which they are supported, even when the type of support is the same.

To explain this facet-dependent sintering behaviour, the researchers further performed DFT calculations for Au_20_ clusters placed on TiO_2_(101) and (001) surfaces. The adsorption energies of Au_20_ clusters on the (001) surface (−2.28 ∼ −3.62 eV) were much larger than those on the (101) surface (−0.30 ∼ −0.47 eV). Also, the difference in adsorption energy of Au_20_ clusters between adsorption sites on the TiO_2_(101) surface was only 0.18 eV, while the value on the TiO_2_(001) surface reached 1.34 eV. Furthermore, the diffusion barriers of the Au adatom on the TiO_2_(101) and (001) surfaces were calculated to be 0.19 eV and 1.31 eV, respectively. Consequently, sintering of Au NPs can occur on the TiO_2_(101) surface by the PMC and OR mechanisms, but is difficult on the TiO_2_(001) surface.

The origin of the remarkable anti-sintering effect of Au NPs on TiO_2_(001) facets can be explained by the domain-matching epitaxial junction between them.^43^ The unit dimensions of the two-dimensional rectangular lattices of Au(111) and A-TiO_2_(001) are 4.995 Å × 2.884 Å and 3.784 Å × 3.784 Å, respectively (Fig. 8E, left). Four times the *d*-spacing of the Au(-110) planes overlaps with three times that of TiO_2_(010) planes, and three times that of Au(11-2) overlaps with four times that of TiO_2_(100) with small mismatches of +1.6% [{(2.884 Å × 4 − 3.784 Å × 3)/3.784 Å × 3} × 100] and −1.0% [{(4.995 Å × 3 − 3.784 Å × 4)/3.784 Å × 4} × 100], respectively (Fig. 8E, right), suggesting the formation of a strong domain-matching heteroepitaxial junction (HEPI) between them. The HEPI junction with the same orientation has recently been reported in the system consisting of Au NPs and A-TiO_2_ nanosheets with dominant (001) surfaces and a thickness of ∼5 nm.^44^

However, most available A-TiO_2_ crystals consist of thermodynamically stable {101} facets rather than the {001} facets.^45^ On the other hand, with R-TiO_2_, NRs can be easily synthesized that have the most stable plane (110) on the side and grow in the *c*-axis direction (the inset in Fig. 1C). The remarkable thermal durability of the Au NPs on the surface of R-TiO_2_ NRs (Fig. 1C) can also be attributed to the domain-matching HEPI junction between them with (111)_Au_//(110)_R-TiO_2__ orientation.^46^ Furthermore, Bokhimi and his collaborators recently reported that Fe doping in R-TiO_2_ improves the stability of Au catalysts, suggesting that this is due to an increase in the number of surface O atoms acting as pinning centers.^47^

## 5 Summary and future challenges

This perspective discussed the fundamentals of Au/MO catalysts, their catalytic activity towards low-temperature CO oxidation and thermal stability, focusing throughout on the nanosize effects of Au NPs. The CTV-DP method utilizing the thermal nano-effect of Au NPs allows their average size to be controlled on different MO supports while maintaining a constant loading. In the Au/MO catalysts, the number of catalytically active sites present on the surface of Au NPs and their perimeter sites increases sharply when *d*_Au_ < 5 nm due to the dimensional effect, while the quantum size effect of Au NPs can also appear when *d*_Au_ < 4 nm. The electronic state of the supported Au NPs can be tuned by electron transfer from the MO support, and further electron transfer to O_2_*via* the Au NPs gives rise to effective reductive activation of O_2_ adsorbed around the Au NPs. This SAO-ET allows the oxidation of CO to proceed smoothly even at low temperatures as a result of removing the spin forbidden properties of the reaction. We have demonstrated that the LH mechanism involving water-assisted SAO-ET as a key step can rationally explain the important findings regarding low-temperature oxidation of CO in the presence of water. Furthermore, the thermal stability of supported Au NCs can be significantly improved by forming an interfacial bond that is matched at the atomic level with the support. As discussed above, the catalytic activity of Au/semiconducting MO catalysts depends on many factors. Focusing specifically on the MO support, selecting a MO with a high conduction band minimum energy, thereby increasing the *E*_F_ level, enables effective O_2_ activation and enhances catalytic activity for low-temperature CO oxidation (Fig. 7B). This information will also be useful for the design of various supported metal NCs other than MOs such as polymers^48^ for low-temperature aerobic oxidation reactions. On the other hand, forming a strong interfacial bond through a heteroepitaxial junction between Au NPs and the MO support can be an effective means of enhancing the thermal stability of the Au NCs (Fig. 1C and 8).

Finally, we present personal views on future challenges and prospects for Au/MO NCs. It has recently been shown that Au/ZrO_2_ exhibits a significantly high activity for CO oxidation (Fig. 6A and B). Since ZrO_2_ is an insulator, the reaction mechanism shown in Fig. 7C cannot be directly applied to these systems. Elucidating this through experimental and theoretical research could lead to the development of novel supported Au NCs. Regardless of the type of target reaction, the greatest challenge in leveraging the excellent catalytic performance of Au NCs is improving their thermal stability, as the sintering can occur at relatively low temperatures (Fig. 1B). The combination of the computational science-aided design of heteroepitaxial junctions between Au NPs and MO supports with the progress in the synthesis technology of faceted MO crystals would offer a promising approach to achieving this objective. Furthermore, supported single-atom catalysts are of great scientific and practical importance because they can exhibit high reactivity and selectivity due to the large degree of coordinative unsaturation and uniformity of the active sites and can also significantly reduce the use of expensive noble metals.^49^ Shedding light on the CO oxidation mechanism over supported single-atom catalysts, which may be significantly different from the Au/MO catalytic reaction mechanism, and the thermal stabilization of single metal atoms on the support surface are important and interesting research topics. We sincerely hope that Au/MO catalysts will find widespread practical applications through rational catalyst material design, grounded in a deep understanding of their reaction mechanisms, that achieves not only high activity and selectivity but also high thermal stability.

## Author contributions

H. T. conceived the perspective, supervised the project and wrote the manuscript. S. N. conducted the literature review, prepared the figures, and performed the proofreading.

## Conflicts of interest

There are no conflicts to declare.

## Data Availability

No primary research results, software or code have been included and no new data were generated or analysed as part of this review.
